# Safety and efficacy of outpatient intravenous diuresis in decompensated heart failure: a systematic review

**DOI:** 10.3389/fcvm.2024.1481513

**Published:** 2024-11-25

**Authors:** Roshni S. Kalkur, John P. Hintz, Girish Pathangey, Katharine A. Manning

**Affiliations:** ^1^ Department of Internal Medicine, Dartmouth-Hitchcock Medical Center, Lebanon, NH, United States; ^2^ Heart and Vascular Center, Dartmouth-Hitchcock Medical Center, Lebanon, NH, United States

**Keywords:** heart failure, diuretics, health services research, diuresis clinic, outpatient intravenous diuresis

## Abstract

**Introduction:**

Heart failure (HF) burdens the US healthcare system, with annual costs exceeding $30 billion. Outpatient intravenous (OP IV) diuresis in clinic or home settings may potentially improve outcomes and reduce costs, though limited data exists. This systematic review evaluates the safety, efficacy, and outcomes of OP IV diuresis in managing decompensated HF as a hospitalization alternative.

**Methods:**

Following PRISMA 2020 guidelines, this systematic review used MeSH terms in MEDLINE, SCOPUS, CINAHL Complete, and Cochrane Central. From 16 selected studies, 15 were single-center; 6 prospective, 9 retrospective; and 1 was a randomized trial comparing OP IV diuresis to oral home regimen. Demographics, visit data, and outcomes were collected, and 30-day outcomes were compared to inpatient IV (IP IV) diuresis from 2021 Medicare HF hospitalizations.

**Results:**

The review included 1,590 unique patients treated with OP IV diuretics, with a mean age of 70 ± 6 years, 69.7% male, and 74.8% NYHA III-IV. Minimal adverse post-diuresis events such as hypokalemia, hypotension, and worsening renal function occurred (4.5%, 0.7%, and 2.3% respectively). Post-visit mean weight loss was −2.2 ± 1.1 kg. The 30-day readmission rate for OP IV diuresis was significantly lower than IP IV diuresis (20.0% vs. 22.6%; *p* = 0.0.401), and 30-day mortality was also lower (5.6% vs. 10.7%; *p* = 0.003).

**Discussion:**

OP IV diuresis is a safe and effective treatment for decompensated HF with minimal risk of adverse events. Data demonstrate reduced 30-day readmission, mortality rates, cost. These findings highlight the potential of OP IV diuresis as an enhanced alternative HF care; however, further randomized control trials are needed to evaluate long-term outcomes.

## Introduction

Heart failure admissions burden the United States healthcare system, with annual costs exceeding $30 billion, and the prevalence of heart failure is projected to surge 50% by 2030 (1). Current heart failure guidelines recommend intravenous (IV) loop diuretics as the primary treatment during exacerbations to reduce volume overload and associated symptoms (2). Traditionally, IV diuretics are administered in the inpatient setting to monitor for adverse effects. The most common adverse effects include electrolyte derangements, metabolic alkalosis, prerenal azotemia, and hypotension. The average hospital length of stay for congestive heart failure in the United States is 4–7 days (3). Transitional care models are being explored as a method of decompressing the burden of heart failure patients in hospitals and preventing unnecessary hospitalizations, which can often lead to further complications. One proposed model is outpatient intravenous (OP IV) diuresis in either the clinic or home setting.

Outpatient IV diuresis has shown potential in managing heart failure exacerbations, by reducing hospital readmissions and costs. It may provide more accessible and affordable treatment options, promoting health equity for socioeconomically disadvantaged heart failure patients (1, 4, 5). Additionally, it could address rural healthcare challenges by improving access and quality of care. This approach has been shown to be feasible as a novel strategy of care to integrate into existing healthcare infrastructures, potentially enhancing patient outcomes and satisfaction and challenging the current inpatient IV diuresis model (4). This systematic review evaluates the safety, efficacy, and outcomes of outpatient IV diuresis in managing decompensated heart failure as an alternative to traditional hospitalization.

## Methods

### Objectives

This systematic review aims to analyze the safety and efficacy of administering IV diuretics in the outpatient setting to patients with an acute heart failure exacerbation when compared to traditional management with inpatient hospitalization. Our search included studies that examined patients with both heart failure with reduced ejection (HFrEF) and heart failure with preserved ejection fraction (HFpEF) that presented with symptoms consistent with worsening heart failure, and that were treated with intravenous diuretics in an outpatient setting. The outcomes of interest were efficacy, measured by readmission rates and average reduction in weight, as well as safety, measured by mortality rates and incidence of adverse events, specifically hypotension, hypokalemia, and acute kidney injury.

### Literature search strategy

A search was performed in the electronic databases MEDLINE, SCOPUS, CINAHL Complete, and Cochrane Central using non-controlled vocabulary search terms and the following controlled vocabulary indexation terms (MeSH and textwords for MEDLINE, Search codes for SCOPUS, and textwords for CINAHL Complete and Cochrane Central): Diuretics, Outpatients, and Intravenous. The search strategy was developed with the support of an information specialist to ensure a comprehensive approach incorporating all key search terms. An overview of the complete search strategy is available upon request. Selection criteria included full-text articles written in English, studies conducted in human adults, and included patients with heart failure and the use of IV diuretics outside of a hospital/inpatient setting. Review studies, case reports, case series, abstracts, studies examining the treatment with intravenous diuretics in the emergency department, or use of subcutaneous or oral diuretics were excluded.

### Study selection and data extraction

**Figure 1:**
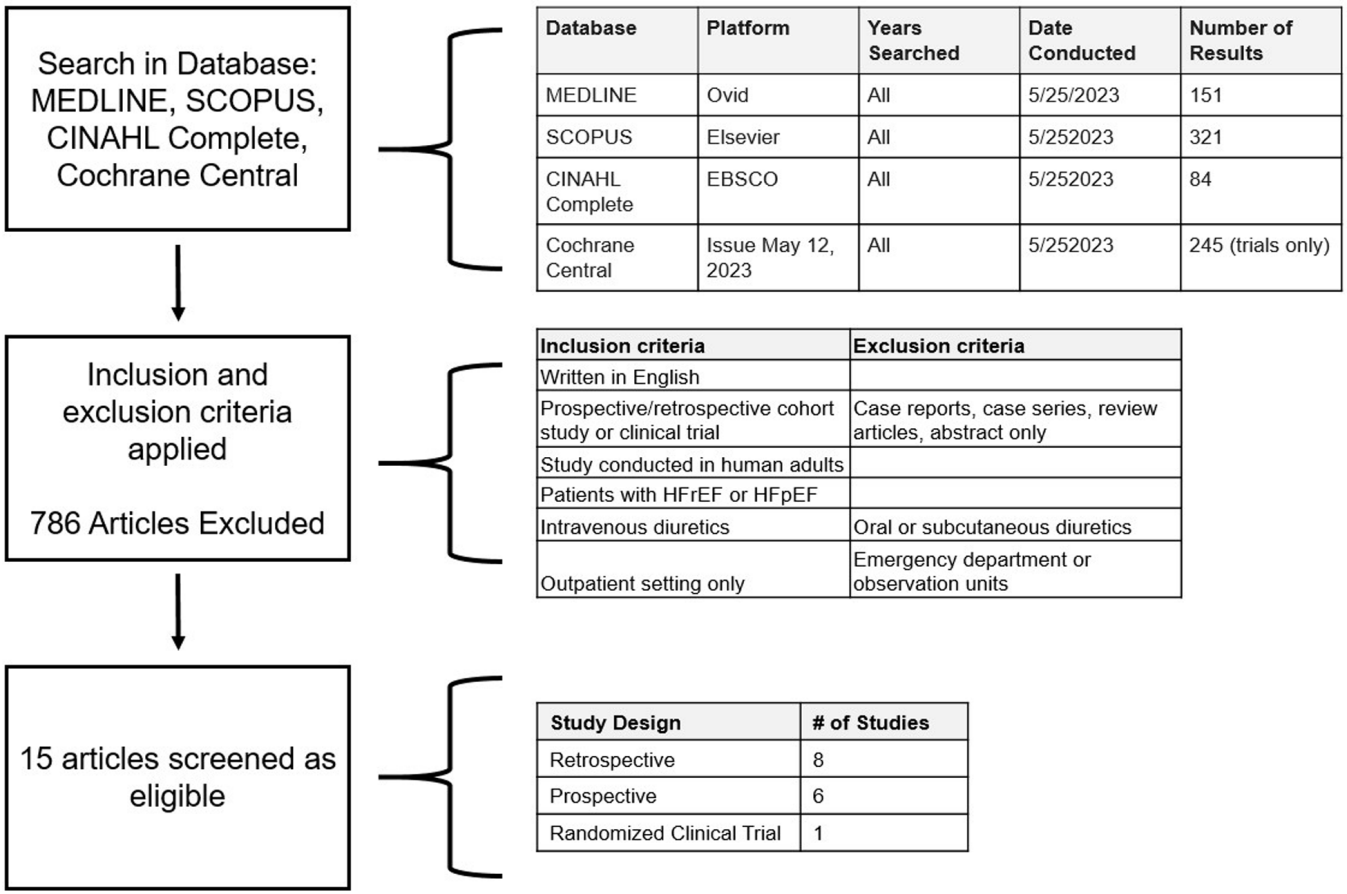
Schematic of search strategy performed in four clinical databases. Inclusion and exclusion criteria for included studies are listed. The search yielded 15 eligible articles, with the majority being either retrospective or prospective studies.

A total of 801 titles and/or abstracts of studies retrieved using the search strategy were independently screened by three reviewing authors to identify studies that potentially met the inclusion criteria. Full texts of relevant studies were retrieved and independently assessed for eligibility. Of these, 786 articles were excluded, and 15 studies were selected to be included in this systematic review for data extraction. See Figure 1 for a flowchart of article selection on eligibility and inclusion of studies for data extraction. Data from selected studies were extracted manually by two authors. Patient characteristics, selection criteria, study protocol and treatment regimens, treatment outcome (all-cause mortality, re-hospitalizations for HF, efficacy, cost, and adverse events), and the limitations of these studies were examined. 30-day all-cause mortality and readmission rates were compared to patients hospitalized for decompensated heart failure and treated with IV diuresis from the 2021 Medicare database. Statistical analysis was conducted using a Chi-square test with Yates' correction.

## Results

This analysis included 15 studies: 14 were single-center, eight were retrospective cohort studies, five were prospective cohort studies, and one was a randomized controlled trial. The studies exhibited considerable variation in selection criteria, baseline characteristics, and treatment design among the studies. Baseline demographics and study designs are reported in Table 1. The average age was 70, with 69.7% being male, and 74.8% classified as NYHA Class III-IV. Common exclusion criteria included severe symptoms, significant comorbid conditions, or hemodynamic instability. The mean ejection fraction varied widely, and almost all studies included patients with both preserved and reduced ejection fractions. Commonly reported co-morbid conditions among patients included atrial fibrillation, coronary artery disease, diabetes mellitus, chronic kidney disease, hypertension, hyperlipidemia, and chronic obstructive pulmonary disease.

**Table 1:** Baseline characteristics of patients from included studies. The number of total patients in the study, along with the subset of patients that received OP IV diuresis is reported. The mean age (in years), gender (percentage male), mean LVEF (percentage), and NYHA classification of patients among each study are reported. Co-morbidities of patients included in the studies are listed. Most studies included the following: atrial fibrillation, coronary artery disease (CAD), diabetes mellitus (DM), chronic kidney disease (CKD), hypertension (HTN), hyperlipidemia (HLD), and chronic obstructive pulmonary disease (COPD).

Study	Number of Patients		Mean Age, yr	Gender (% Male)	Mean LVEF	NYHA Classification	Comorbidities, *n* (%) All Patients						
	Total	Receiving OP IV diuretics					Atrial fibrillation	CAD	DM	CKD	HTN	HLD	COPD
Vivek Verma et al. (6)	27	27	72	93.00%	N/A	III	15 (56%)	13 (48%)	18 (67%)	23 (85%)	18 (67%)	14 (52%)	8 (30%)
Carine Hamo et al. (7)	94	62	64	56.40%	33.50%	III and IV	30 (31.9%)	40 (42.5%)	53 (56.4%)	41 (51.1%)	89 (94.7%)	55 (59.1%)	20 (21.3%)
Leo Buckley et al. (8)	60	60	70	56.70%	N/A	III and IV	29 (48.3%)	31 (51.7%)	31 (51.7%)	28 (46.7%)	46 (76.7%)	N/A	12 (20%)
Kathy Hebert et al. (9)	577	130	57	72.90%	24.00%	III and IV	N/A	N/A	(59 (45.4%)	N/A	106 (81.5%)	N/A	N/A
Girish Pathangey et al. (4)	60	60	70	58.00%	49.00%	III and IV	31 (52%)	30 (50%)	32 (53%)	29 (48%)	41 (68%)	42 (70%)	11 (18%)
Carolyn Rosner et al. (10)	116	116	74	56.50%	50.00%	III and IV	N/A	N/A	52 (44.8%)	N/A	N/A	N/A	N/A
Eric Wierda et al. (11)	259	259	76	62.50%	41.00%	III and IV	171 (66%)	N/A	110 (42.5%)	N/A	169 (65.3%)	N/A	55 (21.2%)
Ilia Halatchev et al. (12)	36	14	70	97.20%	40.00%	III and IV	20 (55.6%)	17 (47.2%)	23 (63.9%)	14 (38.9%)	36 (10%)	N/A	11 (30.6%)
Fozia Ahmed et al. (13)	154	79	77	64.20%	N/A	N/A	92 (63.4%)	78 (53.8%)	68 (46.9%)	98 (67.6%)	93 (64.1%)	N/A	43 (30.0%)
Joban Vaishnav et al. (14)	44	44	71	75.00%	46.00%	III and IV	N/A	N/A	N/A	N/A	N/A	N/A	N/A
A. Ioannou et al. (15)	245	245	73	67.40%	N/A	N/A	109 (44.5%)	106 (43.3%)	92 (37.6%)	163 (66.5%)	133 (54.3%)	N/A	33 (13.5%)
Kamal Alghalayini et al. (16)	105	105	65	73.60%	N/A	N/A	N/A	N/A	95 (90.5%)	N/A	101 (96.2%)	N/A	N/A
Marshall Brinkley et al. (17)	176	176	70	83.60%	40.00%	III and IV	N/A	88 (50.3%)	87 (49.4%)	N/A	N/A	N/A	N/A
Sunal Makadia et al. (18)	247	106	68	53.00%	39.00%	N/A	108 (42.7%)	146 (59.1%)	130 (52.6%)	110 (44.5%)	224 (90.7%)	164 (66.4%)	88 (35.6%)
Mary Ryder et al. (5)	107	107	71	75.00%	38.40%	III and IV	N/A	N/A	36 (33%)	N/A	N/A	N/A	26 (24%)

**Figure 2:**
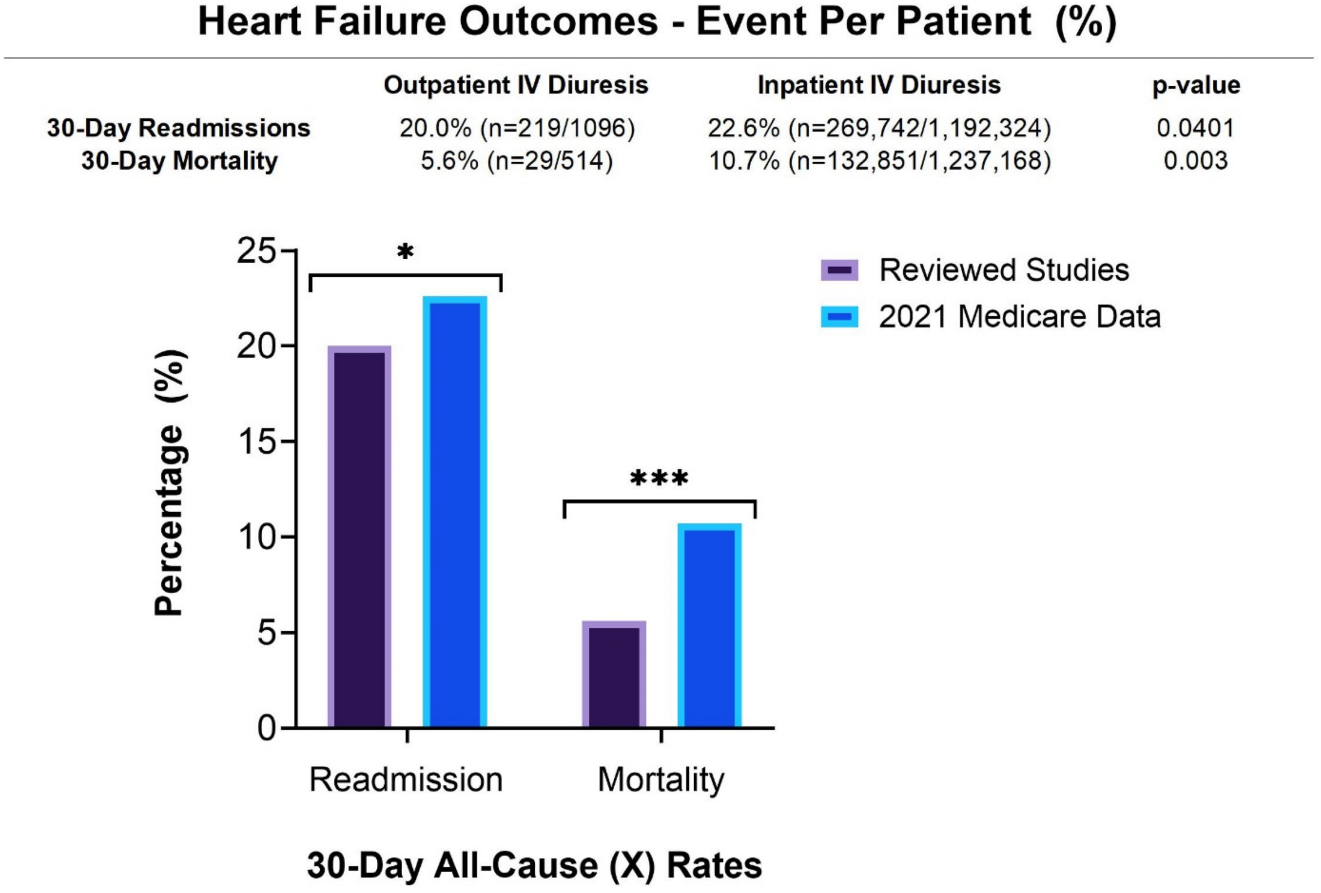
30-day readmission rates and mortality rates among patients who received outpatient IV diuresis in all of the studies combined were compared to 2021 medicare data of patients that received inpatient IV diuresis. 30-day readmission rates (*p* = 0.0401) and 30-day mortality rates (*p* = 0.003) were statistically significant.

A total of 1,590 unique patients were treated with outpatient IV diuretics across these studies. Most studies used a combination of bolus and infusion intravenous furosemide therapy to treat their patients (Table 2). In most studies, dosage was determined by the patient's home diuretic dose. The average dose of IV furosemide given among reported studies was 165.4 mg. Few adverse events were reported, the most common being hypokalemia (4.5%), acute kidney injury (2.3%), and hypotension (0.7%). A majority of studies evaluated efficacy by measuring urine output and change in weight after the infusion. The average urine output among the studies with this reported endpoint was 1,067 ml. The average post-infusion weight loss was 2.2 kg. Most studies analyzed cost savings, although there was great variation in how it was reported. Two studies reported cost savings in Euros (€) rather than USD ($) (Table 2). 13 studies analyzed re-admission rates, 12 studies analyzed mortality rates, and 8 studies analyzed cost savings. The 30-day all-cause readmission rate was 20.0%, and the 30-day all-cause mortality rate among the studies was 5.6%. The average weight reduction following IV diuretics was −2.2 kg. These readmission and mortality rates among the studies were compared to inpatient IV diuresis data from 2021 Medicare HF hospitalizations for heart failure treated with inpatient IV diuresis (Figure 2). The outpatient IV diuresis showed a statistically significant lower 30-day mortality rates (5.6% vs. 10.7%, *p* = 0.003) and 30-day readmission rates (20.0% vs. 22.6%, *p* = 0.0401) compared to 2021 Medicare inpatient data.

**Table 2:** Op IV diuresis treatment strategy for each study is reported. Average IV furosemide dose (mg) among patients in each study is reported. Common adverse side effects such as hypokalemia, hypotension, and AKI are reported for each study. Efficacy was most commonly measured by urine output (ml) and weight change (kg), which is reported for each study. Cost savings for each study was reported with variation between each study, with the most common currency being US dollars ($) or Euros (€).

Study	OPIV Diuretic Strategy, IV furosemide		Safety Outcomes,% of patients receiving OP IV diuresis			Efficacy		Cost savings
	Intervention	Average dose, mg	Hypokalemia	Hypotension	AKI	Average urine output, ml	Average weight change, kg	
Vivek Verma et al. (6)	Bolus + infusion	180	85.2	0	0	1,500	−1.27	$10,395 per patient
Carine Hamo et al. (7)	Bolus + infusion	187	9.7	3.2	14.5	794.5	−2.5	N/A
Leo Buckley et al. (8)	Bolus + infusion +/− thiazide	260	6.7	0	16.7	1,045	−1	N/A
Kathy Hebert et al. (9)	Bolus, infusion, or both	N/A	0	0	0	N/A	N/A	$2,954,586 total
Girish Pathangey et al. (4)	Bolus or infusion	Bolus group: 108, infusion group: 53	5	0	18.3	761	−3.9	$426,111 total
Carolyn Rosner et al. (10)	Bolus	120	0	0	1.7	1,400	N/A	N/A
Eric Wierda et al. (11)	Bolus + infusion	236	0	0.4	0	N/A	N/A	€25M yearly total
Ilia Halatchev et al. (12)	Bolus + infusion +/− thiazide	80	0	0	0	N/A	−1	$839.4 per patient
Fozia Ahmed et al. (13)	Infusion	N/A	1.3	0	1.3	N/A	−3.1	N/A
Joban Vaishnav et al. (14)	N/A	N/A	12	8	2	375	−0.5	$71,047 total
A. Ioannou et al. (15)	Bolus	N/A	N/A	N/A	N/A	N/A	N/A	€2,921 per admission avoided
Kamal Alghalayini et al. (16)	Infusion	N/A	N/A	N/A	N/A	N/A	1,064.57	N/A
Marshall Brinkley et al. (17)	Bolus	160	N/A	N/A	N/A	1,200	N/A	N/A
Sunal Makadia et al. (18)	Bolus +/− metolazone	100	23	0	0	1,460	−2.3	$5,969 per patient per 180 days
Mary Ryder et al. (5)	Bolus	N/A	0	0	1	N/A	−2	N/A

## Discussion

### Safety, efficacy, and outcomes

Based on this evidence, the reviewed studies suggest that outpatient treatment with intravenous diuretics of patients with worsening HF has a low risk of adverse events, primarily AKI (2.3%), hypotension (0.7%), and hypokalemia, with hypokalemia being the most common (4.5%). Almost all studies included regular vital signs and laboratory monitoring before and after the administration of IV diuretics. Adverse events were typically reported as mild and transient. No patients in these studies required escalation of care due to an adverse event. The minority of patients who required readmission shortly after treatment had worsening HF refractory to IV diuresis rather than complications from treatment. Most patients required frequent clinic follow up visits after IV diuresis administration for additional safety monitoring. One study (13) focused exclusively on patients with cardiac amyloidosis and found that outpatient IV diuresis was similarly safe and effective in this population.

The selected studiesprovide evidence for reduction in rates of 30-day readmissions when compared to 2021 Medicare data (20.0% vs. 22.6%, *p* = 0.0401This suggests that outpatient IV diuresis is an acceptable alternative to manage acute heart failure, sparing patients an admission without leading to a rise in the need for readmission. Prior studies, such as the systematic review conducted by Wierda, et al. (2) did not demonstrate any significant reduction in readmission or mortality rates. This suggests that the many advances in heart failure management over the last decade have improved our ability to care for these patients outside of the hospital. Among the individual studies, there has been mixed evidence on the ability of transitional care models to reduce readmission rates for patients with heart failure. Further randomized trials are needed to better elucidate why readmissions occur, and how to prevent them with outpatient interventions.

Interestingly, our collective data also demonstrated a statistically significant decrease in 30-day mortality rates when compared to the 2021 Medicare data of patients hospitalized for heart failure exacerbations (5.6% vs. 10.7%, *p* = 0.0003). This may be explained by limited data and power, with this review incorporating additional studies. Additionally, many selected studies had strict exclusion criteria, which indicates a sampling bias towards patients that have milder disease being selected for outpatient diuresis. Another explanation is that routine complications of hospitalization are being avoided in the outpatient setting such as hospital-acquired pneumonia, venous thromboembolism, line-associated infections, delirium, and other iatrogenic insults. Lastly, advances are being made in the safety and treatment of heart failure itself such as the development of mortality-reducing medications and advanced monitoring techniques. The inability to control the baseline demographics and co-morbid conditions between the studies, and also among patients included in 2021 Medicare data, is a limitation of this study. Further studies are needed to directly compare outpatient and inpatient IV diuresis while controlling for variables such as age, gender, ejection fraction, and co-morbid conditions.

Cost was also analyzed in the majority of studies. The average cost savings per patient ranged from $839.4–$10,395. Based on median data, HF hospitalizations contributed to 65% of all medical HF costs over a 1-year treatment period post hospitalization (19). In addition to readmission, decreasing the incidence of emergency room visits may also play a crucial role in driving cost down. While operating an outpatient clinic requires significant financial resources and staffing, these studies demonstrate a cost benefit when compared to the traditional model of care.

### Clinical decision making and health system considerations

There are many socioeconomic factors that influence whether a patient is hospitalized, regardless of HF severity such as patient preference, access to transportation, caregiver support, and other comorbid conditions that require a higher level of care. In certain patient populations, most often patients in the beginning stages of HF, intravenous diuretics administered in an outpatient setting is a reasonable alternative to traditional inpatient intravenous diuresis. This is also particularly important in a rural healthcare system where improving access to and quality of care are of significance due to costs and time associated with travel (4).

In order to determine which patients would benefit the most from outpatient treatment, further studies are needed to assist in establishing risk stratification and clinical decision making. One potential area of development is to create a risk stratification system to classify patients as low, intermediate, or high-risk based on their co-morbidities, initial vital signs, laboratory values, and severity of heart failure. One study (16) incorporated utilization of spot urine sodium as a triage tool in determining appropriateness of ambulatory IV diuresis. They found that high spot urine output was a useful indicator in generating an acceptable response to outpatient diuresis. These tools may help to triage patients in a more standardized way across institutions. It would also be beneficial to incorporate travel distance and time in considering the most ideal candidates for outpatient IV diuresis.

Prior studies have demonstrated that patients prefer outpatient treatment when compared to inpatient treatment (20). As our healthcare shifts to a more patient-centered approach, we have learned that matching patients' preferences to treatment options and settings may raise treatment adherence and outcomes. Outpatient diuresis also aims to reduce broader costs associated with inpatient hospitalization, and minimize delays in care that are due to over-saturation of emergency departments and inpatient units. Many of the studies included in this review did report cost-associated outcomes, and the general data represents an overall cost improvement with outpatient treatment. It is estimated that in the United States, savings of 650 million to more than 2 billion dollars is possible with a shift towards outpatient treatment for HF (2). This is becoming increasingly important in maintaining access to care as the estimated population of heart failure continues to rise.

Notably, there are logistical challenges related to running outpatient diuresis clinics such as staffing, space, access to diuretic infusions, and equipment needed for close monitoring of vital signs and laboratory values. Further advanced ambulatory techniques mentioned above would also incur further costs for the patients and healthcare systems. Other alternatives to intravenous diuresis are currently being explored, with subcutaneous diuretics as an area of potential interest. This has been shown to have increased bioavailability when compared to oral diuretics, and may be a suitable alternative when outpatient intravenous diuresis is not an option (21). Further studies are ongoing to better elucidate the role of subcutaneous diuretics in the management of acute decompensated heart failure. Randomized control trials with an inpatient IV diuresis control group are needed to more accurately compare safety and outcomes between the outpatient and inpatient setting.

## Conclusions

This systematic review examines the safety and efficacy of outpatient intravenous diuresis to treat heart failure exacerbations. Outpatient IV diuresis appears to be safe, with a relatively low risk of adverse reactions (most commonly hypokalemia, AKI, and hypotension). When compared to inpatient intravenous diuresis Medicare data, there is a statistically significant difference in both 30-day readmission and mortality rates Outpatient diuresis prioritizes quality of life, increases cost savings on both a patient and system-wide level, and may decrease mortality rates compared to inpatient diuresis. Further studies are needed to stratify patients that are the most likely to benefit from outpatient diuresis, to compare the safety profile directly to inpatient IV diuresis, and to investigate the logistical barriers that may prevent patients from accessing care at these clinics in a safe and effective manner.

## Data Availability

The original contributions presented in the study are included in the article/Supplementary Material, further inquiries can be directed to the corresponding author.
